# Multidimensional scaling improves distance-based clustering for microbiome data

**DOI:** 10.1093/bioinformatics/btaf042

**Published:** 2025-01-28

**Authors:** Guanhua Chen, Xinyue Wang, Qiang Sun, Zheng-Zheng Tang

**Affiliations:** Department of Biostatistics and Medical Informatics, University of Wisconsin-Madison, Madison, WI 53726, United States; Department of Statistics, Pennsylvania State University, University Park, PA 16802, United States; Department of Statistical Science, University of Toronto, Toronto, ON M5S 3G3, Canada; Department of Biostatistics and Medical Informatics, University of Wisconsin-Madison, Madison, WI 53726, United States

## Abstract

**Motivation:**

Clustering patients into subgroups based on their microbial compositions can greatly enhance our understanding of the role of microbes in human health and disease etiology. Distance-based clustering methods, such as partitioning around medoids (PAM), are popular due to their computational efficiency and absence of distributional assumptions. However, the performance of these methods can be suboptimal when true cluster memberships are driven by differences in the abundance of only a few microbes, a situation known as the sparse signal scenario.

**Results:**

We demonstrate that classical multidimensional scaling (MDS), a widely used dimensionality reduction technique, effectively denoises microbiome data and enhances the clustering performance of distance-based methods. We propose a two-step procedure that first applies MDS to project high-dimensional microbiome data into a low-dimensional space, followed by distance-based clustering using the low-dimensional data. Our extensive simulations demonstrate that our procedure offers superior performance compared to directly conducting distance-based clustering under the sparse signal scenario. The advantage of our procedure is further showcased in several real data applications.

**Availability and implementation:**

The R package MDSMClust is available at https://github.com/wxy929/MDS-project.

## 1 Introduction

Clustering microbiome data into meaningful subgroups is crucial for understanding the role of microbes in human health and disease etiology. Identifying these clusters serves as a vital step toward leveraging microbiome data for precision medicine. For instance, the concept of enterotypes—distinct clusters of human gut microbiota—was first introduced by Arumugam *et al.* (2011) and subsequently studied by Ding and Schloss (2014) and Costea *et al.* (2018). The 2011 Nature paper by Arumugam *et al.* (2011) identified three enterotypes: Type 1 dominated by *Bacteroides*, Type 2 by *Prevotella*, and Type 3 by *Ruminococcus*. Further research demonstrated that gut microbiome enterotypes are strongly associated with long-term dietary patterns (Wu *et al.* 2011), highlighting their potential for diet-based interventions in precision medicine.

Clustering microbiome data is challenging due to the complex structure of the data. Microbiome data are typically derived from high-throughput sequencing technologies. In amplicon sequencing, specific marker genes, such as the 16S ribosomal RNA gene, are targeted to profile microbial communities. The resulting sequencing reads are processed through bioinformatics pipelines (Bolyen *et al.* 2019) to generate Operational Taxonomic Units (OTUs) or Amplicon Sequence Variants (ASVs) (Callahan *et al.* 2016). Both OTUs and ASVs serve as essential units for downstream statistical analyses, where each sample is represented as a vector of counts corresponding to these units. In this paper, we use “OTU” to represent the analysis unit. The sequencing depth (The total number of reads for a sample) is not correlated with the actual microbial load (the total amount of microorganisms or their DNA volumes) in the sample. Therefore, the counts are compositional data that represent the relative abundance of the OTUs (Gloor *et al.* 2017). In addition, the counts of many OTUs often display zero-inflation patterns due to the proliferation of low-prevalence OTUs and substantial variability across samples (Li 2015). These unique characteristics make microbiome data difficult to model using conventional probabilistic distributions.

Distance-based clustering methods, such as Partitioning Around Medoids (PAM)/k-medoids (Kaufman 1990), are widely employed for microbiome data clustering due to their computational efficiency and lack of reliance on distributional assumptions. These methods use pairwise distances between samples, calculated using metrics specifically designed for microbiome compositions. Different distance metrics can capture distinct aspects of the microbiome data structure, offering flexibility in addressing its unique characteristics.

However, distance-based clustering methods face two major challenges when applied to microbiome data. The distance matrix is typically calculated using all OTUs, including many that contribute little to the clustering signal. This can result in a noisy distance matrix, where signals from a small subset of informative OTUs—often driving the clustering structure—are diluted by noise from uninformative OTUs. This issue, referred to as the sparse signal scenario, can significantly reduce clustering sensitivity and obscure meaningful patterns (Koren *et al.* 2013, Yang and Xu 2020). Furthermore, the meaningfulness of pairwise distances degrades in high-dimensional data. As discussed in Beyer *et al.* (1999), in high-dimensional spaces, the distances between the nearest and farthest neighbors converge, reducing the discriminative power of distance-based clustering methods. This phenomenon is exacerbated in microbiome data, where sparse signals coexist with high dimensionality, making it difficult to uncover meaningful clusters.

Reducing the data to a lower-dimensional space can help denoise the input and alleviate the convergence issues of distances in high-dimensional settings. Therefore, dimensionality reduction techniques offer a promising solution to enhance distance-based clustering performance. A natural choice for the dimensional reduction in microbiome studies is classical multidimensional scaling (MDS) (Borg and Groenen 2005), also known as principal coordinates analysis (PCoA). Unlike principal component analysis (PCA), which operates on Euclidean distances (Martino *et al.* 2019), MDS works directly with distance matrices and can accommodate non-Euclidean distance metrics such as Bray-Curtis or UniFrac that are better suited for compositional microbiome data (Armstrong *et al.* 2022).

Alternative dimensionality reduction techniques like t-distributed stochastic neighbor embedding (t-SNE) (Van der Maaten and Hinton 2008) and Uniform Manifold Approximation and Projection (UMAP) (McInnes *et al.* 2018) can also accommodate non-Euclidean metric. However, these methods often require extensive tuning of multiple parameters, which can compromise reproducibility and stability. In contrast, MDS is computationally simpler, requires fewer tuning decisions, and directly preserves pairwise distances, making it well-suited as a preprocessing step for microbiome data.

In this paper, we propose incorporating MDS as a denoising step prior to performing distance-based clustering. Specifically, high-dimensional microbiome data are projected into a lower-dimensional space using MDS, after which clustering is performed using Euclidean distances in the reduced space. While MDS is widely recognized as a visualization tool, its potential as a preprocessing step to enhance clustering outcomes in microbiome studies remains underexplored. We evaluate the effectiveness of MDS in improving the performance of downstream distance-based clustering methods by addressing the following key questions: (i) Does MDS improve clustering accuracy compared to using the original high-dimensional data? (ii) How do the results depend on the choice of distance metrics, data rarefaction, and methods for selecting the number of clusters? Extensive simulations and real microbiome data applications demonstrate that MDS enhances the robustness and accuracy of distance-based clustering in high-dimensional microbiome datasets, effectively addressing challenges posed by compositionality and sparsity. Our study provides practical guidance for integrating MDS as a preprocessing step to improve clustering performance in microbiome research.

## 2 Materials and methods

Suppose there are *n* samples and *P* OTUs in the dataset. The OTU table can be represented as an *n *×* P* matrix, $X={\left(X_{1},X_{2},\ldots ,X_{n}\right)}^{T}$, where each row $X_{i}={\left(x_{i1},\ldots ,x_{iP}\right)}^{T}$ is a *P*-dimensional vector representing either OTU counts or relative abundances for the *i*-th sample (we abuse the notation to let **X** represent both). The sequencing depth for the *i*-th sample is defined as $N_{i}=\sum_{p=1}^{P}x_{ip}$. Due to the compositional nature of microbiome data, clustering should be performed on relative abundances, as sequencing depth does not accurately represent absolute microbial load. If **X** contains raw counts, it must be normalized to proportions by dividing each row by its corresponding sequencing depth *N_i_*. After normalization, **X** resides in the *P*-dimensional simplex space.

### 2.1 MDS-enhanced distance-based clustering

Classical multidimensional scaling (MDS) is a well-established method for dimensional reduction. The goal of MDS is to project samples from a high-dimensional space into a lower-dimensional space while preserving the distance relationships between them. For example, if sample *A* is closer to sample *B* than to sample *C* in high-dimensional space, MDS aims to maintain this relationship in the reduced space. This technique can be applied to any well-defined distance metric.

To enhance distance-based clustering, we propose first projecting high-dimensional microbiome data into a lower-dimensional space using MDS. It is crucial to use distance metrics designed for microbiome data that account for compositional properties. After projection, the data can be treated as Euclidean, allowing the application of distance-based clustering algorithms such as PAM/k-medoids clustering. The following outlines our algorithm for integrating MDS with PAM/k-medoids clustering.

#### 2.1.1 MDS-enhanced distance-based clustering algorithm

**Input:** The OTU table **X**, represented as an *n *×* P* matrix.

1. **Calculate pairwise distance matrix (Gram matrix).** Calculate the pairwise distance matrix D(X), an *n* × *n* matrix where the element at the *i*-th row and *j*-th column is $d(x_{i},x_{j})$. Here, $d(x_{i},x_{j})$ is defined as the distance between OTUs of the *i*-th and *j*-th samples based on the chosen distance metric *d*.
2. **Apply double centering.** Use the centering matrix $L=I-\frac{11^{T}}{n}$ to calculate $B=-\frac{1}{2}LD(X)L$, ensuring that the row and column means of **B** are all zeros.
3. **Eigen decomposition.** Perform eigen decomposition of **B**: $B=Q\Lambda Q^{T}$, where **Q** is an *n* × *n* orthogonal matrix whose columns are the eigenvectors of **B**, and Λ is an *n* × *n* diagonal matrix with the eigenvalues of **B** on its diagonal.
4. **Construct low-dimensional representation.** Construct the low-dimensional representation $Y=Q_{r}\Lambda_{r}^{1/2}$, an *n* × *r* matrix with rank *r* (a positive integer much smaller than *P*). $Q_{r}$ is composed of the first *r* columns of **Q**, and $\Lambda_{r}$ is an *r* × *r* diagonal matrix consisting of the first *r* rows and columns of Λ. The rank *r* can be determined using the eigenvalue-ratio method (Ahn and Horenstein 2013) or specified by the user. Now, each sample is represented by an *r*-dimensional vector instead of a *P*-dimensional one.
5. **Apply PAM/k-medoids clustering.** Apply k-medoids clustering on **Y** using the Euclidean distance, where the number of clusters *k* is determined by one of the cluster number estimation methods discussed later. Given *k*, let $C=\{c_{1},c_{2},\ldots ,c_{k}\}$ be the set of *k* medoids (one for each cluster), each initially chosen from the *n* samples. The objective is to interactively minimize $\sum_{i=1}^{n}\min_{j\in \{1,\ldots ,k\}}||y_{i}-c_{j}|{|}_{2},$ where $y_{i}$ is the *i*-th row of **Y** and $||y_{i}-c_{j}|{|}_{2}$ is the Euclidean distance between $y_{i}$ and the medoid $c_{j}$. After each iteration, the medoids **C** are updated to reflect the new set of medoids that minimize the total distance, defined as the sum of the distances from each point to its closest medoid. The process continues until the objective function converges, resulting in the final set of *k* medoids $C=\{c_{1}^{\prime },c_{2}^{\prime },\ldots ,c_{k}^{\prime }\}$, where $c_{j}^{\prime }$ is the final medoid representing the *j*-th cluster.
**Output:** The cluster membership vector **M** of length *n* has elements *M_i_*, where $M_{i}\in \{1,\ldots ,k\}$ represents the cluster membership of sample *i*.
  Note that Steps 1–4 essentially constitute the MDS procedure. The described procedure is general and can be applied to any distance metric *d* and distance-based clustering method. For example, k-medoids clustering can be replaced with other distance-based methods, such as k-means (Lloyd 1982).

### 2.2 Distance metrics

Various distance metrics that are compositionality-aware and well-suited for microbiome data are available in open-source R packages such as vegan (Oksanen *et al.* 2015), ade4 (Dray and Dufour 2007), and phyloseq (McMurdie and Holmes 2013). These metrics can be categorized into two types: those that directly account for compositionality and those that transform the *P*-dimensional simplex space to a (P−1)-dimensional Euclidean space, making Euclidean distance applicable post-transformation. Additionally, some distance metrics utilize phylogenetic tree information while others do not, as reviewed in Tang *et al.* (2016) and Shi *et al.* (2022). In our numerical studies, we consider seven representative distance metrics, with their formulas provided in Supplementary Table S1 and their key features briefly described in the following sections.

Bray–Curtis (Bray and Curtis 1957) and Jaccard distances are two popular distance metrics on the simplex space that do not utilize the phylogenetic tree. The Bray–Curtis distance relies on abundance differences, while the Jaccard distance measures differences in the presence-absence of unique OTUs between two samples. UniFrac distances and their extensions incorporate phylogenetic tree information (Lozupone and Knight 2005, Lozupone *et al.* 2007). Unweighted UniFrac uses OTU presence-absence differences between samples to determine branch inclusion, whereas weighted UniFrac incorporates the relative abundance differences to weight branches. The generalized UniFrac further introduces a parameter to reduce the impact of high abundance lineages (Chen *et al.* 2012). For distances built on transformed compositional data, we consider the centered log-ratio (CLR) transform, which does not use phylogenetic tree information, as well as the phylogenetic ILR (PhILR) transform, a variation of the isometric logratio (ILR) transform that uses phylogenetic tree information (Silverman *et al.* 2017).

### 2.3 Cluster number selection methods

Besides the distance, another important decision is selecting the number of clusters, also known as the “k” in k-medoids clustering. We include three commonly used methods: GAP statistic (Tibshirani *et al.* 2001), prediction strength (PS) (Tibshirani and Walther 2005), and Silhouette index (SI) (Rousseeuw 1987). Details of these methods can be found in the Supplementary File S1.

## 3 Results

### 3.1 Simulation settings

We considered a four-cluster problem, with each cluster having a sample size of 100, resulting in a total sample size of 400. We simulated the sequencing depths using a truncated normal distribution with a lower bound of 2, 000, an upper bound of 50, 000, a mean of 10, 000, and a standard deviation of 15, 000. Samples within the same clusters were generated from the same parametric distribution, while parameters were varied for different clusters. We considered two scenarios of parametric distributions for generating the compositional data: Dirichlet multinomial (DM) distribution (Bishop *et al.* 2007), and logistic-normal (LN) multinomial distribution (Mead 1965). Specifically, for the *i*th sample, let $X_{i}$ be the OTU count with a total of *P* OTUs and sequencing depth *N_i_*. The latent cluster membership *Z_i_* for this sample can take discrete values from 1 to 4, representing the four possible clusters.

For the Dirichlet multinomial model (Scenario 1):


$$\begin{array}{c} \pi_{i}=\{\pi_{1},\pi_{2},\ldots ,\pi_{P}\}∼\text{Dirichlet}(\theta_{Z_{i}})\\ X_{i}|\pi_{i}∼\text{Multinomial}(\pi_{i},N_{i}), \end{array}$$

where $\theta_{Z_{i}}$ is a *P*-dimensional vector representing the parameters for the Dirichlet distribution. If two samples (*i* and *j*) belong to the same cluster, they share the same parametric distribution: $\theta_{Z_{i}}=\theta_{Z_{j}}$ if *Z_i_* = *Z_j_*.

For the logistic-normal multinomial model (Scenario 2), additionally denote $y_{i}$ as the logit-transformed proportion vector of the OTUs, then:


$$\begin{array}{c} y_{i}∼N(\mu_{Z_{i}},\Sigma_{Z_{i}}),\;\pi_{i}=\frac{\;\text{exp}(y_{i})}{1+\text{exp}(y_{i})},\\ X_{i}|\pi_{i}∼\text{Multinomial}(\pi_{i},N_{i}) \end{array}$$

where $\mu_{Z_{i}}$ and $\Sigma_{Z_{i}}$ are the mean vector and covariance matrix of the multivariate Gaussian distribution for the *Z_i_*-th cluster. Similar to the Dirichlet Multinomial Model, $\mu_{Z_{i}}=\mu_{Z_{j}}$ and $\Sigma_{Z_{i}}=\Sigma_{Z_{j}}$ if *Z_i_* = *Z_j_*.

#### 3.1.1 Scenario 1

Following Chen *et al.* (2012) and Wu *et al.* (2016), we simulated samples of one group from the Dirichlet multinomial (DM) model with proportion and dispersion parameters estimated from real respiratory tract microbiome data (Charlson *et al.* 2010) via the R package dirmult (Tvedebrink 2010). We partitioned the 856 OTUs into 20 groups using PAM clustering, where OTUs within the same group exhibited similar abundance patterns across all samples. To generate additional clusters, we perturbed two parameters: the proportion parameter (pi) and the dispersion parameter (theta) from the dirmult function.

We considered three settings where differentiation between clusters occurred based on different types of OTU subsets: (1) a *common lineage subset*, (2) a *rare lineage subset*, and (3) a *random OTU subset* consisting of 60 OTUs (20 selected randomly from each of three groups). For the *common lineage subset*, we selected the three most abundant groups (group 6, group 17, and group 7), totaling 73 OTUs, and perturbed their abundance proportions. For the *rare lineage subset*, we selected the three least abundant groups (group 19, group 20, and group 14), which included a total of 17 OTUs, and applied the same perturbation. The same type of perturbation was also applied to the random OTU subset.

In all three settings, the signal strength of perturbation was set to 1.4, with the pi parameter scaled by multiplying it with the signal strength, while the theta parameter remained unchanged. We then simulated counts and corresponding proportions using the Dirichlet multinomial model via the dirmult function.

#### 3.1.2 Scenario 2

We simulated samples from the LN model with mean and covariance estimated from the same respiratory tract microbiome data. Specifically, we first converted the observed OTU counts to proportions and then selected the last OTU as the reference, conducting a logit transformation of the proportional data. To ensure the validity of the logit transformation, we added 0.5 to the OTU counts.

We then fitted a multivariate Gaussian distribution to the transformed data. Using the maximum likelihood estimator, we estimated the mean vector and covariance matrix of this multivariate Gaussian distribution. Given these parameters, we simulated data and transformed them back to proportions. Finally, we generated the count data using a multinomial distribution with the simulated proportions.

To simulate differential OTUs for the other three clusters, we altered the mean of the underlying multivariate Gaussian distribution by multiplying it by factors of 2.75, 5.8, and 3.6 for *common lineage subset*, *rare lineage subset*, and *random OTU subset*, respectively. Differentiation occurred in one of the three OTU subsets described in Scenario 1.

It is important to note that while we focused on specific subsets of OTUs to introduce differentiation signals, the full dataset comprised 856 OTUs, resulting in a high-dimensional setting where *P *=* *856 exceeds *n *=* *400.

We compared PAM clustering with and without the MDS step under different distance metrics and cluster number selection methods. As a benchmark, we also included a popular model-based clustering approach, the Dirichlet multinomial mixture method (DMM) (Holmes *et al.* 2012). The number of clusters for DMM was set to 4, which is the true number of clusters.

In summary, the true distribution of OTUs in Scenario 1 was a Dirichlet Multinomial mixture with four components, while in Scenario 2, it was a Logistic-Normal Multinomial mixture with four components. We compared PAM with and without the MDS step using the distances listed in Section 2.2. Results are presented both under the true number of clusters (oracle) and under the estimated number of clusters determined by one of the three cluster estimation methods listed in Section 2.3. Rarefaction is a common preprocessing step to address differences in sequencing depth by subsampling reads to the same depth. To evaluate its impact on clustering, OTU counts were either rarefied or kept non-rarefied before being normalized to proportions and used for clustering analysis. Each simulation was repeated for 100 times.

Since the underlying true cluster membership was known for every sample, we evaluated the performance of different clustering methods against the true cluster membership using the adjusted Rand index (ARI) (Hubert and Arabie 1985). ARI measures the agreement between two cluster memberships, with a value ranging from –1 to 1. A value of 1 indicates a perfect match, 0 indicates a purely random match, and –1 indicates a complete mismatch.

The aforementioned main simulation study focused on settings with weak cluster separation, where achieving good clustering is inherently challenging. For completeness, we also conducted simulations for the strong signal setting (Supplementary Section C2). Moreover, besides the MDS, we also conducted simulations to evaluate the alternative dimensionality reduction techniques, including PCA, t-SNE, and UMAP (Supplementary Section C3).

### 3.2 Simulation results

**Figure 1.**
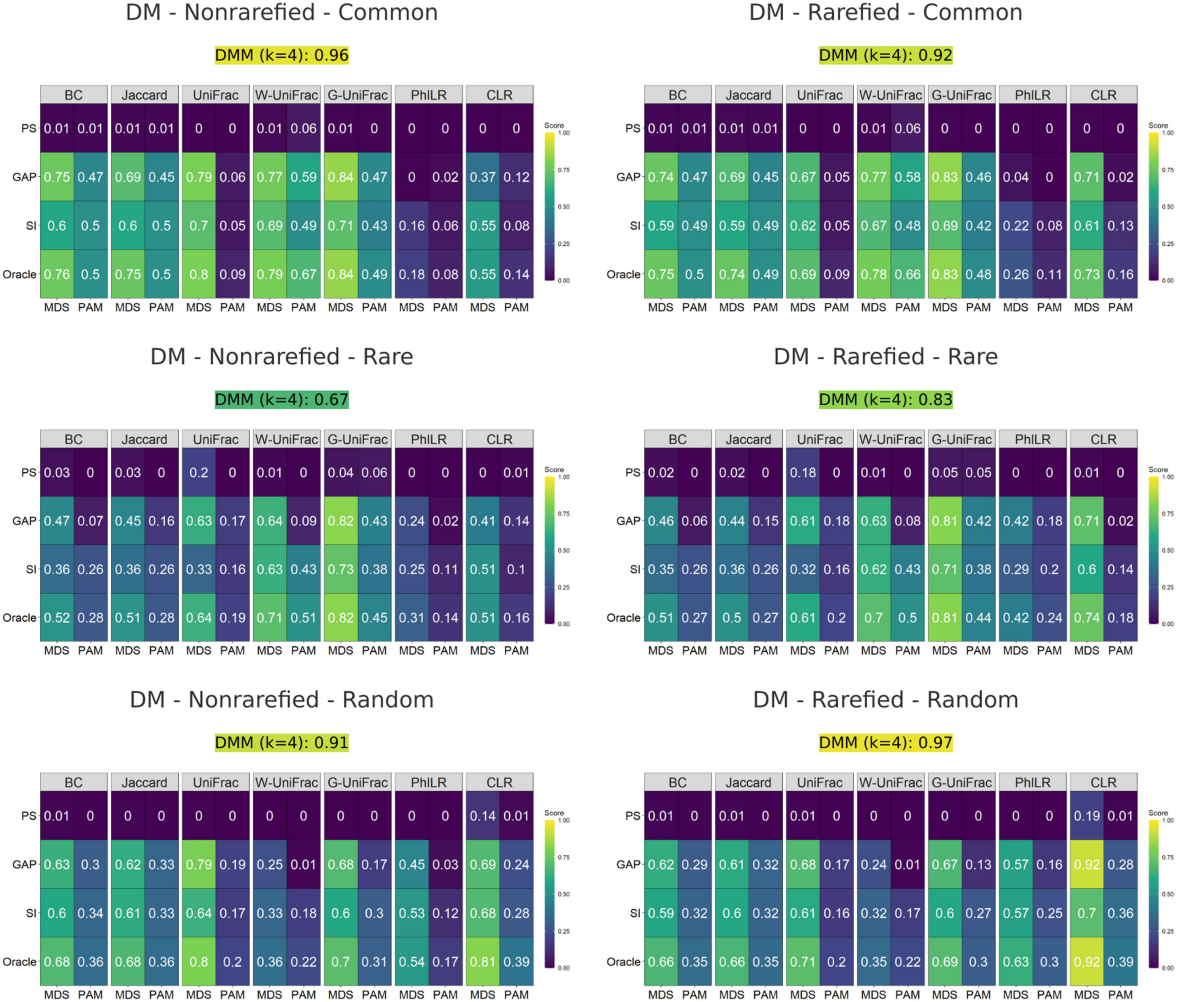
ARI of different clustering methods for Scenario 1 with DM as the generative model. The clustering results with non-rarefied OTU data are on the left, and those with rarefied OTU data are on the right. Within each heatmap, different distance metrics are displayed in columns, and cluster number selection methods are displayed in rows. MDS and its PAM counterpart are placed side by side. The DMM model performance is included in the subcaption. Brighter colors indicate better performance.

**Figure 2.**
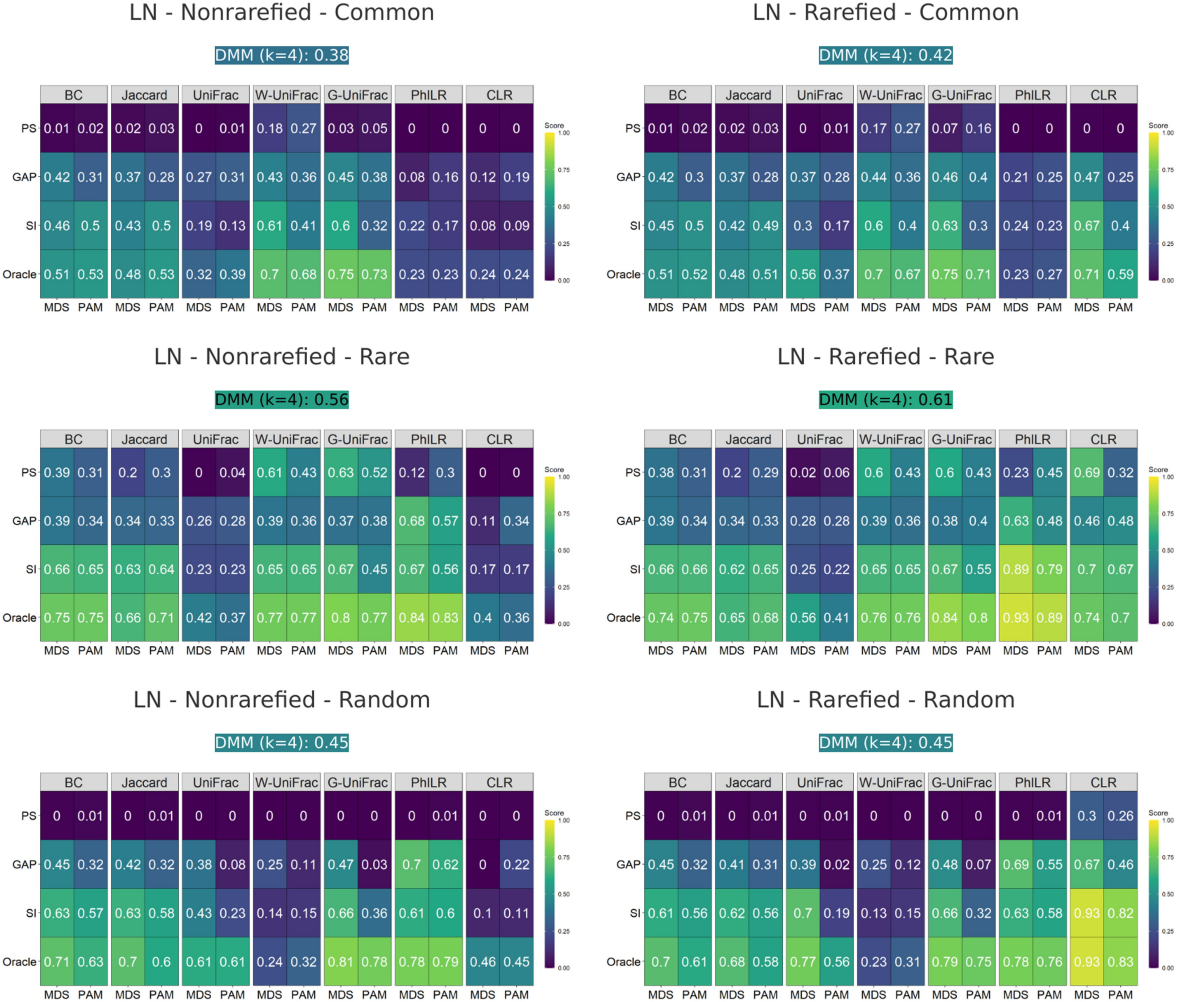
ARI of different clustering methods for Scenario 2 with LN as the generative model. The clustering results with non-rarefied OTU data are on the left, and those with rarefied OTU data are on the right. Within each heatmap, different distance metrics are displayed in columns with the cluster number selection methods displayed in rows. MDS and its PAM counterpart are placed side by side. The DMM model performance is included in the subcaption. Brighter colors indicate better performance.

For both scenarios, MDS improved the distance-based clustering results as shown in Figs 1 and 2. When the oracle number of clusters was provided, MDS performed better than its PAM counterpart for all distance metrics. Moreover, when the number of clusters needed to be estimated, which is the most common situation in practice, the advantage of MDS over PAM was even more substantial. Compared to DMM, distance-based clustering was more robust to the underlying data-generating mechanism, especially when the MDS step was included. Specifically, the Dirichlet multinomial mixture model was the true model for Scenario 1 and a mis-specified model for Scenario 2; hence, the DMM method performed very well in Scenario 1 for all settings but poorly in Scenario 2. In contrast, MDS performed relatively well in both scenarios.

For both scenarios, the SI and Gap statistics worked well (see Supplementary Tables S2–S13). When the data were from a logistic-normal multinomial mixture, SI could better select the number of clusters for MDS. When the data were from a Dirichlet multinomial mixture, Gap statistics could better select the number of clusters. Prediction strength did not perform well, as it always underestimated the number of clusters in our simulations with the recommended cutoff point, and it was unclear how the cutoff should be tuned in practice.

When comparing the results of the left panels to the right panels of Figs 1 and 2, MDS with transformation-based metrics (PhILR and CLR) showed more variation in performance between rarefied and non-rarefied data, indicating higher sensitivity to rarefaction compared to MDS with non-transformation-based metrics (other metrics). Furthermore, DMM also seemed to be affected by rarefaction, though this was not consistent across all scenarios. For example, when signals were added to the rare OTUs under Scenario 1, the ARI of DMM without rarefaction was 0.67, while the ARI of DMM with rarefaction was 0.83. In contrast, when signals were added to the random OTUs under Scenario 2, the ARI of DMM with or without rarefaction were both 0.45.

Additionally, we conducted analyses under strong cluster membership settings, with results presented in Supplementary Fig. S1. In most cases, MDS outperformed or matched PAM, with both methods achieving an ARI close to 1, reflecting the strong clustering signal. However, MDS underperformed compared to PAM when GAP was used as the cluster number selection criterion paired with Jaccard or UniFrac distance metrics. Unlike in weak cluster membership settings, PS performed well as a cluster selection method under strong signals. SI consistently demonstrated reliable performance, while GAP showed poor results for certain distance metrics. Overall, SI emerged as the most reliable cluster selection method, performing consistently well across both strong and weak clustering signal settings, while GAP and PS demonstrated inconsistent performance.

We also conducted an analysis using PCA (limited to Euclidean distance) and t-SNE or UMAP (applicable to all seven non-Euclidean distances) for dimensional reduction under Scenario 1. The results are presented in Supplementary Fig. S2. PCA performed poorly, as expected, due to its reliance on Euclidean distance, which is unsuitable for compositional microbiome data. While t-SNE and UMAP demonstrated better performance than PCA, they generally underperformed compared to MDS across almost all scenarios, except when the PS was used for cluster selection. However, PS itself performed less effectively than GAP and SI criteria in Scenario 1. In conclusion, MDS consistently outperformed t-SNE and UMAP, demonstrating greater robustness and accuracy for improving distance-based clustering across the considered scenarios.

### 3.3 Real data applications

We applied our clustering methods to three distinct datasets to evaluate their performance. The first two datasets (denoted as Martinez data and Smits data) consist of samples from different geographical locations, with these locations treated as the underlying true clusters. The third dataset is derived from the NIH Human Microbiome Project (HMP). All the real data applications use non-rarified data as input.

#### 3.3.1 Martinez data

Martínez *et al.* (2015) analyzed a dataset consisting of samples from the Asaro and Sausi communities in Papua New Guinea, as well as from the United States. This dataset contains microbiome information from 62 samples and 10 227 OTUs. These 62 samples are divided into two geolocations: Papua New Guinea (40 samples) and the USA (22 samples). Their study concluded that westernized and non-westernized fecal microbiomes differed substantially. In particular, Papua New Guineans harbored communities with vastly different abundance profiles compared to US residents. For this dataset, the clustering signal appears to be very strong. As shown in Table 1, DMM, as well as many MDS or PAM methods, can fully recover the two geo-location clusters (ARI = 1). Correspondingly, the selected number of clusters for those methods was 2 (see Supplementary Table S14).

**Table 1.** ARI results for Martinez.

	BC		Jaccard		UniFrac		W-UniFrac		G-UniFrac		PhILR		CLR	
	MDS	PAM	MDS	PAM	MDS	PAM	MDS	PAM	MDS	PAM	MDS	PAM	MDS	PAM
PS	1.00	0.81	1.00	0.00	1.00	1.00	0.00	0.00	0.00	0.00	0.00	0.00	1.00	0.94
GAP	0.25	0.73	0.35	0.81	1.00	1.00	0.65	0.70	1.00	1.00	0.43	0.94	1.00	0.94
SI	1.00	0.81	1.00	0.81	1.00	1.00	0.64	0.70	1.00	1.00	0.70	0.94	1.00	0.94

For PS, MDS achieved near-perfect clustering across several distance metrics, including BC, Jaccard, and UniFrac, while PAM showed varied performance with ARIs ranging from 0.00 to 0.94. Notably, PS performed well when paired with both MDS and PAM, particularly under strong cluster separation settings, as observed in the Martinez dataset and validated through simulations for the strong signal setting (Supplementary Section C2).

For Gap Statistics, MDS generally performed similar to or worse than PAM, with the Jaccard distance showing a particularly pronounced difference where PAM outperformed MDS (also observed in the additional simulations in Supplementary Section C2).

For SI, MDS consistently maintained near-perfect clustering across multiple metrics, whereas PAM’s performance was slightly lower, with ARIs ranging from 0.64 to 0.94.

#### 3.3.2 Smits data

Smits *et al.* (2017) longitudinally collected stool samples from Hadza hunter-gatherers of Tanzania, which may exhibit annual cyclic reconfiguration of the microbiome. Following the preprocessing procedure in the R package MicrobiomeCluster (Shi *et al.* 2022), we focused on clustering the samples from the two most distinct seasons: the late dry and the early wet seasons. We had a total of 259 samples, including 62 samples from the early wet season and 197 samples from the late dry season. The dataset contains 12 000 OTUs. For this dataset, the clustering signal appears to be weak. Only a few metrics (five with MDS and four with PAM) combined with the SI could effectively recover the cluster membership (ARI > 0.5). Among these, MDS-CLR with SI performed the best, while other methods showed limited effectiveness, as summarized in Table 2. This is partially due to PS underestimating the number of clusters and GAP overestimating the number of clusters regardless of the actual cluster number. The number of clusters detected by SI was closer to 2 (see Supplementary Table S15). Moreover, the optimal number of clusters identified by DMM using the Laplace approximation was 3 (with an associated ARI = 0.32), and the ARIs of DMM for other values of *k* were lower than 0.32.

**Table 2.** ARI results for Smits.

	BC		Jaccard		UniFrac		W-UniFrac		G-UniFrac		PhILR		CLR	
	MDS	PAM	MDS	PAM	MDS	PAM	MDS	PAM	MDS	PAM	MDS	PAM	MDS	PAM
PS	0.00	0.00	0.00	0.00	0.00	0.00	0.00	0.00	0.00	0.00	0.00	0.00	0.00	0.00
GAP	0.15	0.08	0.20	0.08	0.19	0.08	0.14	0.14	0.33	0.08	0.00	0.00	0.18	0.07
SI	0.66	0.61	0.36	0.61	0.37	0.77	0.64	0.10	0.69	0.64	0.50	0.13	0.84	0.31

#### 3.3.3 HMP data

**Figure 3.**
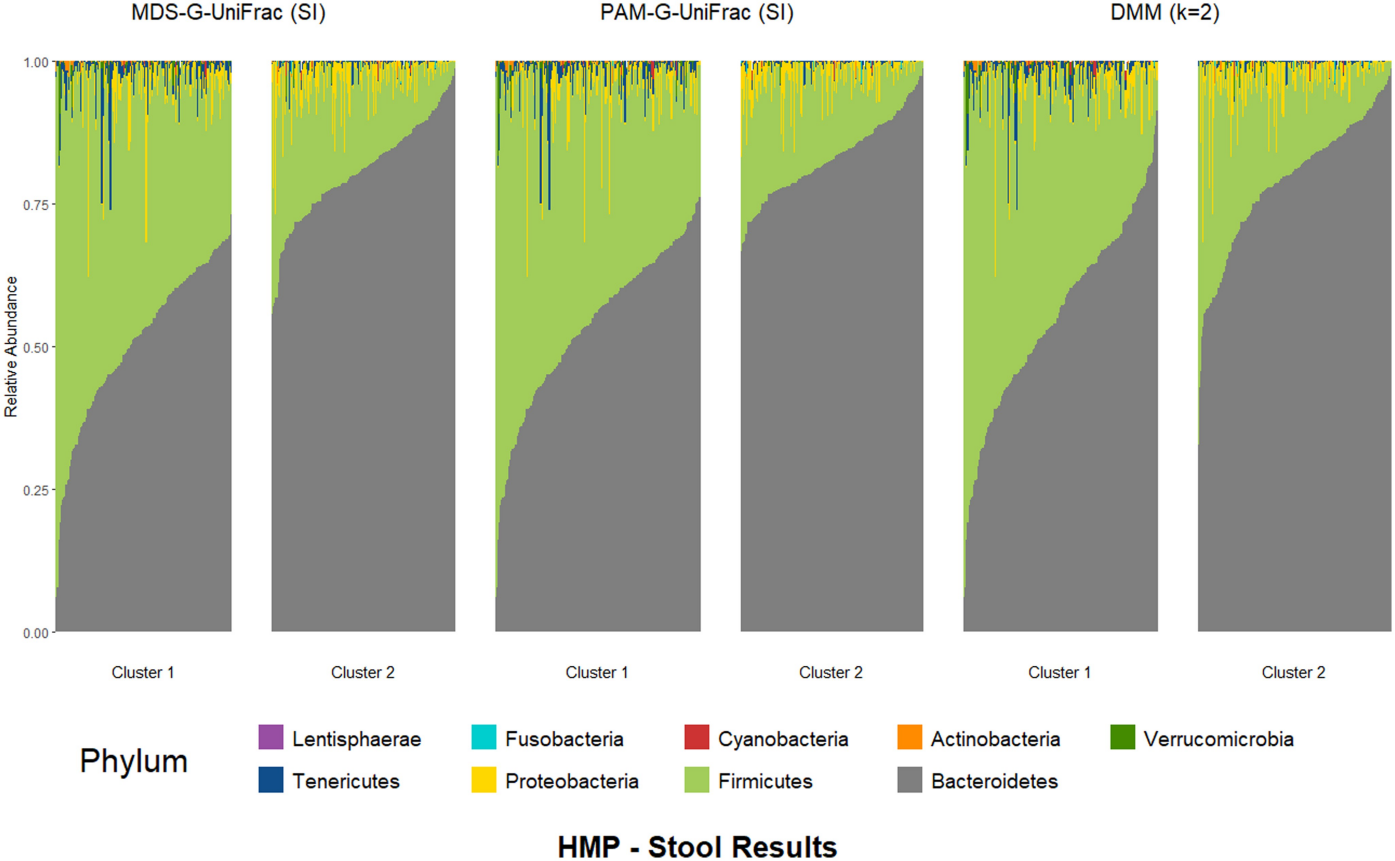
Relative abundance of the top 10 most abundant phyla for HMP stool samples, stratified by identified clusters (enterotypes) using different clustering methods.

We accessed the 16S rRNA sequencing data of variable regions 3–5 from the Human Microbiome Project (Huttenhower *et al.* 2012) via the HMP16SData package (Schiffer *et al.* 2019). The HMP Phase I study sequenced microbes from different body sites of healthy individuals, and we focused on stool samples to identify enterotypes of the gut microbiome. The dataset contains 4743 samples with 45 336 OTUs, including 319 stool samples. We filtered out extremely rare OTUs (total abundance less than 50 across all samples), resulting in 3989 OTUs. Most MDS methods with the SI selected two clusters, while GAP estimated clusters number between 1 to 15. PS concluded there is only one cluster (Supplementary Table S16). The DMM model also identified two clusters, with Laplace suggesting *k* = 2 as the optimal number of clusters, while AIC and BIC suggested *k* = 1 as optimal. Basically, our analysis suggested two enterotypes of the human gut microbiome, with Bacteroidetes and Firmicutes as differentially expressed phyla between clusters (see Supplementary Table S17). Furthermore, Fig. 3 shows the top 10 most abundant phyla and their relative abundance in each sample, grouped by cluster membership according to MDS-GUnifrac (SI), PAM-GUnifrac (SI), and DMM. It is evident that cluster memberships from MDS-GUnifrac and PAM-GUnifrac are driven by the abundance of Bacteroidetes, while the driving phyla for DMM clustering results are less clear.

## 4 Discussion

We proposed a framework using MDS to enhance the performance of distance-based clustering, particularly PAM clustering. Our results show that incorporating MDS improves the robustness of distance-based clustering across different data-generating mechanisms. Overall, the Silhouette Index (SI) is recommended for selecting the number of clusters, as it consistently performs well across both strong and weak clustering signal scenarios and demonstrates robust performance across multiple distance metrics. In contrast, GAP and PS exhibit inconsistent performance across different conditions. In addition, we observed that rarefaction can impact clustering performance for some metrics but not for all.

The effectiveness of MDS stems from its ability to denoise data by projecting samples into a lower-dimensional space, thereby preserving essential structures for clustering. In high-dimensional datasets, the meaningfulness of pairwise distances often degrades, as distances between the nearest and farthest neighbors converge, reducing the discriminative power of clustering methods. MDS addresses this issue by transforming the data into a lower-dimensional space where meaningful patterns and groupings become more discernible. This process also facilitates cluster selection methods, such as the SI, to more accurately determine the number of clusters, especially when clusters are challenging to separate. After the MDS step, the data is no longer compositional; therefore, Euclidean distances can be directly computed between projected data points, aligning with the SI’s reliance on Euclidean distance. Consequently, applying the SI post-projection may yield more accurate cluster estimation than applying it to the original high-dimensional space.

We also applied PCA, t-SNE, and UMAP to real data using geolocation as the true cluster label (Supplementary Section D2). Both t-SNE and UMAP showed inferior performance compared to MDS. Based on results from both simulations (Supplementary Section C3) and real data analysis, we conclude that t-SNE and UMAP are less effective than MDS for enhancing distance-based clustering in microbiome data. One possible reason is that t-SNE and UMAP involve additional complex transformations beyond the original distance matrix during dimensionality reduction (Supplementary Section C3), while MDS directly operates on the Gram matrix of the distance. Additionally, t-SNE and UMAP require extensive tuning of multiple parameters, whereas MDS operates without such complexity.

In this study, we focused on classical MDS; however, generalized versions, such as Metric Multidimensional Scaling (mMDS), exist (Borg and Groenen 2005). Notably, Kernel PCA has been shown to be equivalent to mMDS (Williams 2002), offering a broader framework for dimensionality reduction. Given the demonstrated benefits of classical MDS in our approach, it is reasonable to hypothesize that other types of mMDS could further enhance clustering performance—an open question for future research.

While model-based clustering following dimensionality reduction is an intriguing direction, our study specifically prioritized distance-based clustering. Future work could also focus on developing more accurate methods for determining the optimal number of clusters, particularly tailored to the unique characteristics of microbiome data. Additionally, as no single distance metric universally outperforms others, combining multiple distance metrics may provide more robust and reliable clustering results (Shi *et al.* 2022).

## Supplementary Material

btaf042_Supplementary_Data
