# Supplementary material for: Multidimensional scaling improves distance-based clustering for microbiome data
Source: Bioinformatics. 2025 Jan 28;41(2):btaf042. doi: 10.1093/bioinformatics/btaf042 (PMC11814494; doi:10.1093/bioinformatics/btaf042)
Supplement: btaf042_Supplementary_Data [file btaf042_supplementary_data.zip › MDS_supp_final.pdf]

Supplementary Materials for "Multidimensional scaling improves distance-based clustering for microbiome data"

Guanhua Chen, Xinyue Wang, Qiang Sun, Zheng-Zheng Tang

A Summary of distance metrics designed for compositional data

Distance	Type	Computational Formula		Use Tree
Bray-Curtis	Original	$d^B_{jk} = \frac{\sum_p \lvert x_{jp}-x_{kp}\rvert}{\sum_p (x_{jp}+x_{kp})}$		No
Jaccard	Original	$d^J_{jk} = \frac{2d^{B*}_{jk}}{1+d^{B*}_{jk}}$, where $d^{B*}_{jk} = \frac{\sum_p \lvert I(x_{jp}>0)-I(x_{kp}>0)\rvert}{\sum_p (I(x_{jp}>0)+I(x_{kp}>0))}$		No
Unweighted UniFrac	Original	$d^{(\alpha)}_{jk} = \frac{\sum_p b_p(\tau_{jp}+\tau_{kp})^\alpha \frac{\lvert\tau_{jp}-\tau_{kp}\rvert}{\tau_{jp}+\tau_{kp}}}{\sum_p b_p(\tau_{jp}+\tau_{kp})^\alpha}$	$\alpha = 0$	Yes
Weighted UniFrac	Original		$\alpha = 1$	Yes
Generalized UniFrac	Original		$\alpha = 0.5$	Yes
PhILR-Euclidean	Transformed	Phylogenetic ILR Transformation		Yes
CLR-Euclidean	Transformed	$d^C_{jk} = (\hat{\mathbf{X}}_j - \hat{\mathbf{X}}_k)^T(\hat{\mathbf{X}}_j - \hat{\mathbf{X}}_k)$, where $\hat{\mathbf{X}}_j = \left\{\log\left(\frac{x_{j1}}{G(\mathbf{X}_j)}\right), \cdots, \log\left(\frac{x_{jP}}{G(\mathbf{X}_j)}\right)\right\}$, $G(\mathbf{X}_j) = [\prod_{p=1}^P x_{jp}]^{\frac{1}{P}}$		No

Table S.1: Distance metric summary, assuming $\mathbf{X}$ has already been normalized to proportions.

B Review of cluster number selection methods for distance-based clustering

Gap Statistic: The Gap statistic compares the total intra-cluster variation for different values of k with their expected values under a null reference distribution of the data. Let W_k be the pooled within-cluster sum of squares. The Gap statistic is defined as follows (Tibshirani *et al.*, 2001): $\text{Gap}_n(k) = E_n\{\log(W_k)\} - \log(W_k)$,

where $E_n \log(W_k)$ is the expected value of the logarithm of the within-cluster sum of squares under a null reference distribution. The k corresponding to the maximum Gap statistic is the optimal number of clusters.

Prediction Strength: The "prediction strength" assesses how many clusters can be predicted from the data and how well they can be predicted. If we split the dataset into training and testing sets, we want to select the number of components k so that the k-clustering results can accurately predict samples in the test set. Prediction strength values decrease with k and take the value 1 when $k = 1$. In our simulation and data applications, we set a threshold of 0.9 and determine the optimal number of clusters as the maximum one with prediction strength above this cutoff as suggested by (Tibshirani and Walther, 2005).

Silhouette Index: The silhouette index evaluates the degree of cluster cohesion and dispersion (Rousseeuw, 1987). For each data point $i \in \Omega_i$ (Ω_i is the cluster containing data point i), we have: $s(i) = \frac{b(i)-a(i)}{\max\{a(i),b(i)\}}$ where $a(i) = \frac{\sum_{j\in\Omega_i, j\neq i} d(\mathbf{X}_i, \mathbf{X}j)}{|\Omega_i|-1}$ is the mean distance between the data point i and other data points in Ω_i, which measures within-cluster dispersion. $b(i) = \min_k \frac{\sum j\in\Omega_k d(\mathbf{X}_i, \mathbf{X}_j)}{|\Omega_k|-1}$ is the minimum mean distance between the data point i and all points in any other cluster, measuring between-cluster dispersion. The silhouette index ranges from -1 to 1, with higher values indicating a higher degree of agreement within the cluster and greater separation between clusters. If most data points have relatively high values, the clustering results are considered appropriate. In practice, we provide a range for the cluster number k in advance and select the k that maximizes $\frac{1}{n}\sum_{i=1}^{n} s(i)$.

C Additional simulation settings and results

C.1 Additional simulation results for the weak signal setting

The following tables show the average estimated number of clusters for different clustering methods, considering various combinations of distance metrics and cluster number selection methods.

Table S.2: Estimated Number of Clusters for Scenario 1 (Common, Nonrarefied)

	**BC**		**Jaccard**		**UniFrac**		**W-UniFrac**		**G-UniFrac**		**PhILR**		**CLR**	
	**MDS**	**PAM**	**MDS**	**PAM**	**MDS**	**PAM**	**MDS**	**PAM**	**MDS**	**PAM**	**MDS**	**PAM**	**MDS**	**PAM**
PS	1.026	1.050	1.044	1.034	1.000	1.000	1.048	1.198	1.018	1.022	1.000	1.000	1.000	1.066
GAP	4.086	4.592	4.792	5.186	3.970	3.146	4.130	5.588	4.000	4.374	1.022	1.782	3.474	10.348
SI	3.020	2.984	3.026	2.978	3.406	2.110	3.498	2.734	3.434	2.850	5.686	2.050	3.998	2.016

Table S.3: Estimated Number of Clusters for Scenario 1 (Common, Rarefied)

	**BC**		**Jaccard**		**UniFrac**		**W-UniFrac**		**G-UniFrac**		**PhILR**		**CLR**	
	**MDS**	**PAM**	**MDS**	**PAM**	**MDS**	**PAM**	**MDS**	**PAM**	**MDS**	**PAM**	**MDS**	**PAM**	**MDS**	**PAM**
PS	1.024	1.042	1.040	1.042	1.000	1.000	1.032	1.216	1.016	1.014	1.000	1.000	1.010	1.000
GAP	4.094	4.460	4.784	5.194	3.798	2.548	4.122	5.654	4.000	4.344	1.174	1.024	3.824	1.866
SI	3.020	2.984	3.026	2.978	3.192	2.134	3.438	2.710	3.372	2.842	4.450	2.068	3.204	2.270

Table S.4: Estimated Number of Clusters for Scenario 1 (Rare, Nonrarefied)

	**BC**		**Jaccard**		**UniFrac**		**W-UniFrac**		**G-UniFrac**		**PhILR**		**CLR**	
	**MDS**	**PAM**	**MDS**	**PAM**	**MDS**	**PAM**	**MDS**	**PAM**	**MDS**	**PAM**	**MDS**	**PAM**	**MDS**	**PAM**
PS	1.112	1.002	1.126	1.000	1.608	1.002	1.038	1.006	1.134	1.262	1.000	1.000	1.004	1.098
GAP	4.852	2.876	5.172	4.942	3.060	2.790	4.838	1.772	4.000	4.198	2.006	1.710	4.826	11.404
SI	2.588	2.876	2.768	2.878	2.026	2.110	3.440	3.018	3.474	2.694	2.132	2.082	3.100	2.018

Table S.5: Estimated Number of Clusters for Scenario 1 (Rare, Rarefied)

	**BC**		**Jaccard**		**UniFrac**		**W-UniFrac**		**G-UniFrac**		**PhILR**		**CLR**	
	**MDS**	**PAM**	**MDS**	**PAM**	**MDS**	**PAM**	**MDS**	**PAM**	**MDS**	**PAM**	**MDS**	**PAM**	**MDS**	**PAM**
PS	1.092	1.000	1.118	1.004	1.560	1.012	1.030	1.002	1.168	1.236	1.006	1.004	1.028	1.000
GAP	4.822	3.042	5.222	4.930	3.112	2.854	4.856	1.744	3.996	4.292	2.976	2.700	3.668	1.786
SI	2.568	2.884	2.748	2.884	2.016	2.130	3.416	3.066	3.442	2.718	2.128	2.266	2.972	2.384

Table S.6: Estimated Number of Clusters for Scenario 1 (Random,Nonrarefied)

	**BC**		**Jaccard**		**UniFrac**		**W-UniFrac**		**G-UniFrac**		**PhILR**		**CLR**	
	**MDS**	**PAM**	**MDS**	**PAM**	**MDS**	**PAM**	**MDS**	**PAM**	**MDS**	**PAM**	**MDS**	**PAM**	**MDS**	**PAM**
PS	1.024	1.010	1.012	1.008	1.012	1.000	1.000	1.000	1.000	1.000	1.000	1.002	1.434	1.068
GAP	4.484	5.118	4.870	4.928	3.922	3.392	2.636	1.174	3.746	2.838	2.682	2.006	5.974	13.386
SI	3.154	2.896	3.268	2.880	3.054	2.458	3.206	2.552	3.030	2.912	2.970	2.190	3.058	2.390

Table S.7: Estimated Number of Clusters for Scenario 1 (Random, Rarefied)

	**BC**		**Jaccard**		**UniFrac**		**W-UniFrac**		**G-UniFrac**		**PhILR**		**CLR**	
	**MDS**	**PAM**	**MDS**	**PAM**	**MDS**	**PAM**	**MDS**	**PAM**	**MDS**	**PAM**	**MDS**	**PAM**	**MDS**	**PAM**
PS	1.022	1.012	1.030	1.018	1.018	1.000	1.000	1.000	1.002	1.000	1.000	1.000	1.578	1.038
GAP	4.498	5.060	4.804	5.004	3.714	3.364	2.678	1.166	3.704	2.510	3.014	2.798	4.000	4.604
SI	3.154	2.896	3.268	2.880	3.014	2.432	3.166	2.532	3.028	2.840	2.980	2.600	3.086	2.774

Table S.8: Estimated Number of Clusters for Scenario 2 (Common, Nonrarefied)

	**BC**		**Jaccard**		**UniFrac**		**W-UniFrac**		**G-UniFrac**		**PhILR**		**CLR**	
	**MDS**	**PAM**	**MDS**	**PAM**	**MDS**	**PAM**	**MDS**	**PAM**	**MDS**	**PAM**	**MDS**	**PAM**	**MDS**	**PAM**
PS	1.034	1.074	1.084	1.142	1.002	1.044	1.622	1.934	1.122	1.168	1.000	1.000	1.008	1.056
GAP	7.418	13.812	8.998	14.864	12.038	6.640	10.896	13.986	10.520	12.662	1.696	3.444	3.700	4.130
SI	5.346	4.926	5.866	5.210	3.432	2.004	5.102	3.054	3.940	2.142	2.476	2.252	2.000	2.000

Table S.9: Estimated Number of Clusters for Scenario 2 (Common, Rarefied)

	**BC**		**Jaccard**		**UniFrac**		**W-UniFrac**		**G-UniFrac**		**PhILR**		**CLR**	
	**MDS**	**PAM**	**MDS**	**PAM**	**MDS**	**PAM**	**MDS**	**PAM**	**MDS**	**PAM**	**MDS**	**PAM**	**MDS**	**PAM**
PS	1.028	1.076	1.080	1.150	1.008	1.076	1.596	1.958	1.236	1.570	1.000	1.000	1.008	1.000
GAP	7.468	13.762	9.000	14.806	10.652	11.690	10.696	13.900	10.466	12.652	2.780	3.844	9.106	11.120
SI	5.346	4.926	5.866	5.210	3.178	2.014	5.166	2.992	4.060	2.098	2.644	2.254	3.800	2.688

Table S.10: Estimated Number of Clusters for Scenario 2 (Rare, Nonrarefied)

	**BC**		**Jaccard**		**UniFrac**		**W-UniFrac**		**G-UniFrac**		**PhILR**		**CLR**	
	**MDS**	**PAM**	**MDS**	**PAM**	**MDS**	**PAM**	**MDS**	**PAM**	**MDS**	**PAM**	**MDS**	**PAM**	**MDS**	**PAM**
PS	2.322	2.016	1.910	2.016	1.018	1.166	2.880	2.350	2.892	2.592	1.350	1.962	1.000	1.026
GAP	13.208	15.316	14.842	15.576	12.936	13.600	14.650	15.394	13.852	14.240	6.648	9.328	4.236	7.378
SI	4.322	3.264	5.564	3.976	2.080	2.072	3.068	3.004	3.000	2.400	3.012	2.790	2.000	2.000

Table S.11: Estimated Number of Clusters for Scenario 2 (Rare, Rarefied)

	**BC**		**Jaccard**		**UniFrac**		**W-UniFrac**		**G-UniFrac**		**PhILR**		**CLR**	
	**MDS**	**PAM**	**MDS**	**PAM**	**MDS**	**PAM**	**MDS**	**PAM**	**MDS**	**PAM**	**MDS**	**PAM**	**MDS**	**PAM**
PS	2.330	2.028	1.908	2.004	1.094	1.260	2.876	2.346	2.808	2.332	1.720	2.456	2.964	2.096
GAP	13.266	15.376	14.822	15.598	13.472	11.782	14.568	15.324	13.890	13.636	8.258	11.706	10.150	9.414
SI	4.322	3.264	5.564	3.976	2.092	2.002	3.086	3.004	3.000	2.674	3.832	3.584	3.000	3.238

Table S.12: Estimated Number of Clusters for Scenario 2 (Random, Nonrarefied)

	**BC**		**Jaccard**		**UniFrac**		**W-UniFrac**		**G-UniFrac**		**PhILR**		**CLR**	
	**MDS**	**PAM**	**MDS**	**PAM**	**MDS**	**PAM**	**MDS**	**PAM**	**MDS**	**PAM**	**MDS**	**PAM**	**MDS**	**PAM**
PS	1.014	1.042	1.006	1.036	1.002	1.032	1.008	1.018	1.002	1.004	1.002	1.026	1.000	1.032
GAP	11.008	14.968	12.098	15.402	12.768	3.504	10.020	5.658	11.250	1.874	4.774	7.604	1.026	3.470
SI	5.966	6.364	6.128	6.882	4.654	2.444	2.584	2.844	5.280	2.698	3.002	3.018	2.110	2.000

Table S.13: Estimated Number of Clusters for Scenario 2 (Random, Rarefied)

	**BC**		**Jaccard**		**UniFrac**		**W-UniFrac**		**G-UniFrac**		**PhILR**		**CLR**	
	**MDS**	**PAM**	**MDS**	**PAM**	**MDS**	**PAM**	**MDS**	**PAM**	**MDS**	**PAM**	**MDS**	**PAM**	**MDS**	**PAM**
PS	1.024	1.042	1.004	1.052	1.016	1.034	1.006	1.022	1.008	1.008	1.012	1.042	1.940	1.932
GAP	11.260	14.902	12.244	15.456	8.954	1.912	9.992	6.260	10.786	3.228	5.500	9.128	8.106	12.130
SI	5.966	6.364	6.128	6.882	4.736	2.046	2.590	2.728	5.328	2.460	3.108	3.038	3.994	3.958

C.2 Simulations for the strong signal setting

We generated simulated data with a strong clustering signal by modifying Scenario 1 (Dirichlet multinomial model) from the common lineage type described in the main text. Specifically, we set the signal strength to 10 (increased from 1.4 in the main text), scaling the `pi` parameter while keeping the `theta` parameter unchanged. All other settings remained consistent with Scenario 1. Each simulation was repeated 100 times.

Figure S.1 shows the performance of MDS and PAM in this strong signal setting, with and without rarefaction, where clusters are well separated. Both MDS and PAM, when provided with the oracle number of clusters, achieve near-perfect clustering results. Similar to the weak signal scenario, MDS generally outperforms PAM, while PhILR and CLR remain more sensitive to rarefaction. The Silhouette Index (SI) continues to perform reliably as a cluster selection method. A key difference is that PS performs exceptionally well in this strong signal scenario, particularly PS+MDS, in contrast to its poor performance in the weak signal case. However, GAP+MDS struggles with certain metrics, such as Jaccard, and underperforms compared to GAP+PAM when using UniFrac.

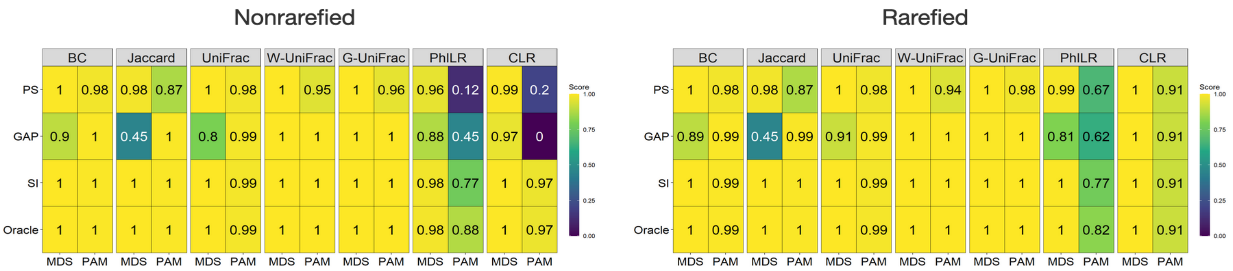

Figure S.1: ARI of different clustering methods for Scenario 1 of common OTUs with DM as the generative model, the signal is stronger than those in the main text. The clustering results with non-rarefied OTU data are on the left, and those with rarefied OTU data are on the right. Within each heatmap, different distance metrics are displayed in columns with the cluster number selection methods displayed in rows. MDS and its PAM counterpart are placed side-by-side.

C.3 Comparison of MDS and PCA, t-SNE, UMAP on Scenario 1 (weak signal setting).

Instead of using MDS as a dimensionality reduction tool, other techniques such as PCA, t-SNE (Van der Maaten and Hinton, 2008), and UMAP (McInnes *et al.*, 2018) can also be applied. In this comparison, steps 1–4 from our workflow were replaced with these techniques, while step 5—clustering in the reduced space—remained unchanged. Below, we provide a brief description of each method.

Principal Component Analysis (PCA): PCA is a linear dimensionality reduction method designed to transform a high-dimensional dataset into a lower-dimensional representation while preserving as much variance as possible. Given an OTU table represented as an $n \times P$ matrix $\mathbf{X}$, PCA projects the data onto a set of orthogonal axes, called principal components (PCs), that maximize the variance of the data. Mathematically, PCA aims to solve the following transformation:

$$\mathbf{Y} = \mathbf{X}\mathbf{W}$$

where: $\mathbf{Y}$ is the $n \times r$ matrix of projected data in the reduced space ($r \ll P$), $\mathbf{W}$ is a $P \times r$ matrix whose columns are the eigenvectors corresponding to the top r eigenvalues of the covariance matrix $\mathbf{\Sigma} = \frac{1}{n-1}\mathbf{X}^T\mathbf{X}$.

The principal components represent directions in the original high-dimensional space along which the data exhibit the most variance. While PCA is computationally efficient and interpretable, it assumes Euclidean distances, which may not accurately represent relationships in non-Euclidean or compositional datasets.

t-Distributed Stochastic Neighbor Embedding (t-SNE): t-SNE is a nonlinear dimensionality reduction technique primarily used for visualizing high-dimensional data in a low-dimensional space (Van der Maaten and Hinton, 2008). Unlike PCA, t-SNE focuses on preserving the local neighborhood relationships between data points rather than maximizing global variance.

Given an OTU table represented as an $n \times P$ matrix $\mathbf{X}$, t-SNE models the pairwise similarities between samples using conditional probabilities. The similarity between two samples i and j in the original high-dimensional space

is defined as:

$$p_{j|i} = \frac{\exp\left(-d(x_i, x_j)/2\sigma_i^2\right)}{\sum_{k\neq i}\exp\left(-d(x_i, x_k)/2\sigma_i^2\right)}$$

where $d(x_i, x_j)$ represents the distance between samples i and j and σ_i is a local scaling parameter controlled by the perplexity hyperparameter.

The joint probability distribution in the high-dimensional space is then symmetrized as: $p_{ij} = \frac{p_{j|i}+p_{i|j}}{2n}$

In the low-dimensional space, with points $\mathbf{y}_i$ and $\mathbf{y}_j$, t-SNE defines a corresponding joint probability distribution using a Student's t-distribution with one degree of freedom:

$$q_{ij} = \frac{\left(1 + \|\mathbf{y}_i - \mathbf{y}_j\|^2\right)^{-1}}{\sum_{k\neq l}\left(1 + \|\mathbf{y}_k - \mathbf{y}_l\|^2\right)^{-1}}$$

where $\|\mathbf{y}_i - \mathbf{y}_j\|$ represents the Euclidean distance between points in the low-dimensional embedding space.

t-SNE aims to minimize the mismatch between the high-dimensional and low-dimensional probability distributions by minimizing the Kullback-Leibler (KL) divergence:

$$KL(P\|Q) = \sum_{i\neq j} p_{ij} \log \frac{p_{ij}}{q_{ij}}$$

This optimization is performed using gradient descent to adjust the positions of points in the low-dimensional space, aiming to preserve local data structures effectively.

While t-SNE excels at capturing local neighborhood relationships and identifying clusters, it relies on several hyperparameters (e.g., perplexity, learning rate) and can struggle to preserve global structures in the data. Additionally, its stochastic nature may lead to variations in results across multiple runs Cai and Ma (2022).

Uniform Manifold Approximation and Projection (UMAP): UMAP is another nonlinear dimensionality reduction technique designed to balance the preservation of local and global structures in high-dimensional data

(McInnes *et al.*, 2018). Given an OTU table represented as an $n \times P$ matrix $\mathbf{X}$, UMAP constructs a weighted graph representation of the data. The probability of an edge between two samples i and j is defined as:

$$p_{ij} = \exp\left(-\frac{\max(0, d(x_i, x_j) - \rho_i)}{\sigma_i}\right)$$

where: $d(x_i, x_j)$ represents the distance between samples i and j, ρ_i is the distance to the nearest neighbor of i, and σ_i is a local scaling parameter.

In the low-dimensional embedding, UMAP optimizes the graph layout by minimizing the cross-entropy loss:

$$C = \sum_{i,j} p_{ij} \log \frac{p_{ij}}{q_{ij}} + (1 - p_{ij}) \log \frac{1 - p_{ij}}{1 - q_{ij}}$$

where q_{ij} represents the low-dimensional equivalent of the graph probabilities.

UMAP preserves both local neighborhood structures and global relationships more effectively than t-SNE. However, it still relies on hyperparameter tuning, and the results may vary depending on the chosen parameters.

When applying those methods to our simulations, for PCA, which inherently works with Euclidean distances, we applied it directly to the simulated count data without prior centering or scaling. The Euclidean distances were then calculated based on the first five principal components (PCs) derived from PCA. For t-SNE and UMAP, distance metrics that are compositionality-aware can be utilized. The t-SNE model was configured to produce a two-dimensional output, adhering to the method's constraints that limit the dimensionality setting to values between 1 and 3. Other t-SNE parameters were set to default. The t-SNE analysis was performed using the R package `Rtsne` (Krijthe and van der Maaten, 2015). In contrast, the UMAP model was implemented with an output dimensionality of five components, while keeping other parameters at default settings. he UMAP analysis was conducted using the R package `uwot` (Melville, 2024). These methods were applied to Scenario 1, and each simulation was repeated 100 times. The quality of the resulting clusters was then assessed using the ARI.

Figure S.2 illustrates the performance differences between MDS, PCA, t-SNE, and UMAP in Scenario 1. Across all scenarios, MDS consistently outperformed PCA, t-SNE, and UMAP, demonstrating superior clustering accuracy and robustness with or without rarefaction. When the oracle number of clusters was provided, PCA consistently failed, as expected, while MDS outperformed both t-SNE and UMAP, with UMAP performing slightly better than t-SNE. This result highlights that the projected data from MDS better preserves the underlying cluster patterns compared to other methods. When the cluster numbers were estimated using PS, GAP, and SI, MDS consistently outperformed t-SNE and UMAP under both GAP and SI criteria. Notably, the performance advantage of MDS under GAP was comparable to that observed under the oracle number of clusters, while the advantage under SI was less pronounced. In summary, when comparing MDS with PAM, the advantage stems from both higher projection quality and better estimation of cluster numbers. In contrast, when comparing MDS with t-SNE or UMAP, the primary advantage comes from the superior quality of the projection provided by MDS.

DM - Nonrarefied - Common

PCA Result

	PS	GAP	SI	Oracle
PCA	0.02	0.41	0.532	0.629

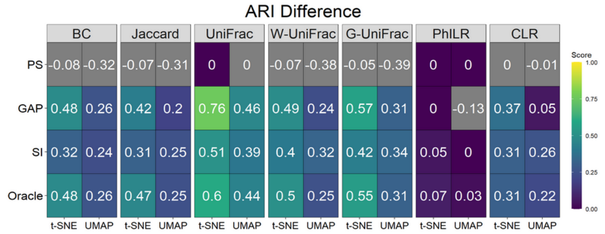

DM - Rarefied - Common

PCA Result

	PS	GAP	SI	Oracle
PCA	0.018	0.397	0.524	0.618

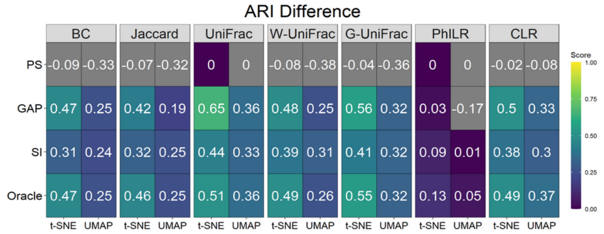

DM - Nonrarefied - Rare

PCA Result

	PS	GAP	SI	Oracle
PCA	0	0.008	0.228	0.368

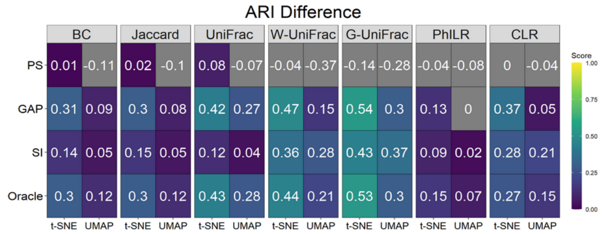

DM - Rarefied - Rare

PCA Result

	PS	GAP	SI	Oracle
PCA	0	0.007	0.229	0.36

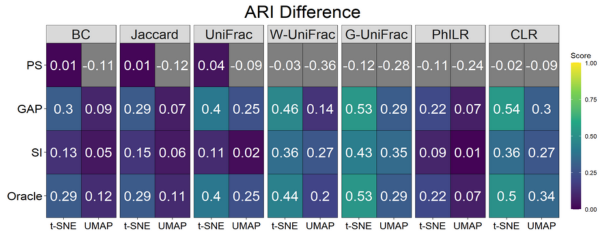

DM - Nonrarefied - Random

PCA Result

	PS	GAP	SI	Oracle
PCA	0.001	0.061	0.293	0.404

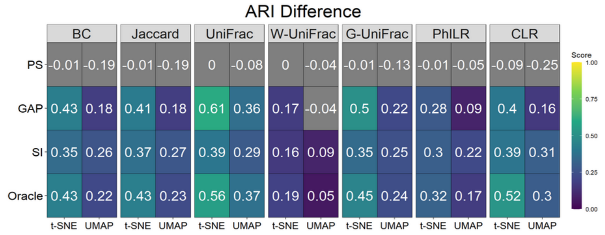

DM - Rarefied - Random

PCA Result

	PS	GAP	SI	Oracle
PCA	0	0.058	0.289	0.394

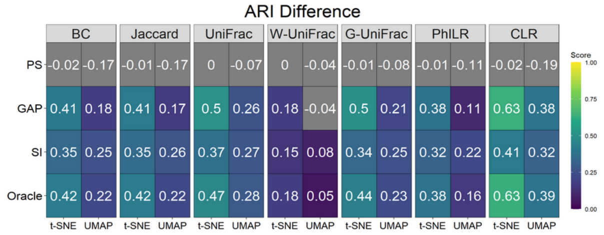

Figure S.2: Comparison of MDS, PCA, t-SNE, and UMAP in Scenario 1 using the Dirichlet Multinomial model as the generative framework. PCA results are presented using absolute ARI values, while t-SNE and UMAP results are shown as ARI differences relative to MDS (MDS - t-SNE or MDS - UMAP). Results for non-rarefied OTU data are displayed on the left, and rarefied OTU data on the right. In each heatmap, distance metrics are represented by columns, and cluster number selection methods by rows. Grey indicates scenarios where t-SNE or UMAP outperformed MDS.

D Additional data application results

D.1 Additional data application results of MDS and DMM

The following tables display the estimated number of clusters for different clustering methods across three real data applications, as well as the differential abundance test results of the top 10 most abundant genera in HMP data, contrasting clusters identified within each of the three methods: MDS-G-Unifrac (SI), PAM-G-Unifrac (SI), and DMM.

Table S.14: Estimated Number of Clusters for Martinez data

	**BC**		**Jaccard**		**UniFrac**		**W-UniFrac**		**G-UniFrac**		**PhILR**		**CLR**	
	**MDS**	**PAM**	**MDS**	**PAM**	**MDS**	**PAM**	**MDS**	**PAM**	**MDS**	**PAM**	**MDS**	**PAM**	**MDS**	**PAM**
PS	2	2	2	1	2	2	1	1	1	2	1	1	2	2
GAP	10	3	6	2	2	2	2	2	2	2	3	2	2	2
SI	2	2	2	2	2	2	3	2	2	2	2	2	2	2

Table S.15: Estimated Number of Clusters for Smits data

	**BC**		**Jaccard**		**UniFrac**		**W-UniFrac**		**G-UniFrac**		**PhILR**		**CLR**	
	**MDS**	**PAM**	**MDS**	**PAM**	**MDS**	**PAM**	**MDS**	**PAM**	**MDS**	**PAM**	**MDS**	**PAM**	**MDS**	**PAM**
PS	1	1	1	1	1	1	1	1	1	1	1	1	1	1
GAP	8	16	6	16	6	16	8	8	3	15	1	1	7	16
SI	2	2	4	2	3	2	2	2	2	2	2	2	2	4

Table S.16: Estimated Number of Clusters for HMP data

	**BC**		**Jaccard**		**UniFrac**		**W-UniFrac**		**G-UniFrac**		**PhILR**		**CLR**	
	**MDS**	**PAM**	**MDS**	**PAM**	**MDS**	**PAM**	**MDS**	**PAM**	**MDS**	**PAM**	**MDS**	**PAM**	**MDS**	**PAM**
PS	1	1	1	1	1	1	1	1	1	1	1	1	1	1
GAP	8	15	6	15	4	14	8	5	9	3	1	1	2	1
SI	5	2	6	2	3	2	2	2	2	2	2	2	2	2

D.2 Comparison of MDS and PCA, t-SNE on Martinez and Smits Data

We also applied PCA and t-SNE for clustering the Martinez and Smits Data. For PCA, the first five principal component was used. For t-SNE, we adjusted the perplexity parameter to 20 for the Martinez dataset to meet the constraint $3 \times \text{perplexity} < n - 1$, where n is the sample size. This adjustment was necessary because Martinez

Table S.17: T-test p-values for the differential abundance of the top 10 most abundant genera in HMP data, contrasting clusters identified within each of the three methods: MDS-G-Unifrac (SI), PAM-G-Unifrac (SI), and DMM. No. OTU is the number of OTUs aggregated to the genus.

**Phylum**	**Genus**	**No. OTU**	**MDS_G-UniFrac**	**PAM_G-UniFrac**	**DMM (k=2)**
Bacteroidetes	Bacteroides	1706	5.6e-47	1.1e-44	0.020
Bacteroidetes	Prevotella	214	0.83	0.47	0.0075
Bacteroidetes	Parabacteroides	164	0.021	0.021	0.067
Firmicutes	Faecalibacterium	146	3.1e-16	3.8e-20	0.036
Firmicutes	Ruminococcus	131	3.8e-09	3.5e-11	0.00080
Bacteroidetes	Alistipes	117	0.012	9.3e-06	0.32
Firmicutes	Oscillospira	101	2.0e-15	3.9e-15	0.30
Firmicutes	Roseburia	78	5.4e-09	1.2e-11	0.32
Firmicutes	Subdoligranulum	70	7.5e-09	1.3e-07	0.11
Tenericutes	Clostridium	61	0.00072	3.5e-05	0.21

has a sample size of 62. For the Smits dataset (sample size: 259), we used the default perplexity setting of 30. All other parameters remained consistent across analyses. However, UMAP analysis could not be conducted due to technical difficulties related to matrix operations in R.

In the Martinez dataset, PCA performed poorly, with ARI values of zero across PS and GAP, while SI achieved a modest ARI of 0.631. In contrast, MDS consistently outperformed both PCA and t-SNE across most distance metrics and cluster selection methods, particularly under PS and SI. The SI demonstrated the most reliable performance, yielding high ARI values across nearly all metrics. GAP and PS showed more variability, with GAP+MDS generally outperforming GAP+t-SNE, except under certain distance metrics like Jaccard.

In the Smits dataset, overall clustering performance was much weaker due to a weaker clustering signal. PCA performed poorly, with ARI values of zero for PS and GAP, and 0.045 for SI. MDS performed slightly better than t-SNE, especially under SI and GAP methods. The PS method failed entirely across all metrics for both MDS and t-SNE. SI remained the most reliable cluster selection criterion in this dataset, though its ARI values were significantly lower compared to those observed in the Martinez dataset.

In summary, MDS outperformed PCA and t-SNE as a dimensionality reduction tool in these data applications, consistent with the findings from simulations presented in Section C.3.

Table S.18: t-SNE ARI results for Martinez (MDS's ARI results are listed for reference purposes)

	**BC**		**Jaccard**		**UniFrac**		**W-UniFrac**		**G-UniFrac**		**PhILR**		**CLR**	
	**MDS**	**tSNE**	**MDS**	**tSNE**	**MDS**	**tSNE**	**MDS**	**tSNE**	**MDS**	**tSNE**	**MDS**	**tSNE**	**MDS**	**tSNE**
PS	1.00	0.42	1.00	0.88	1.00	0.10	0.00	0.70	0.00	0.60	0.00	0.05	1.00	0.70
GAP	0.25	0.42	0.35	0.88	1.00	0.10	0.65	0.00	1.00	0.60	0.43	0.00	1.00	0.70
SI	1.00	0.42	1.00	0.88	1.00	0.10	0.64	0.70	1.00	0.60	0.70	0.05	1.00	0.70

Table S.19: t-SNE ARI results for Smits (MDS's ARI results are listed for reference purposes)

	**BC**		**Jaccard**		**UniFrac**		**W-UniFrac**		**G-UniFrac**		**PhILR**		**CLR**	
	**MDS**	**tSNE**	**MDS**	**tSNE**	**MDS**	**tSNE**	**MDS**	**tSNE**	**MDS**	**tSNE**	**MDS**	**tSNE**	**MDS**	**tSNE**
PS	0.00	0.00	0.00	0.00	0.00	0.00	0.00	0.00	0.00	0.00	0.00	0.00	0.00	0.00
GAP	0.15	0.26	0.20	0.26	0.19	0.00	0.14	0.25	0.33	0.32	0.00	0.08	0.18	0.00
SI	0.66	0.26	0.36	0.27	0.37	0.13	0.64	0.25	0.69	0.33	0.50	0.08	0.84	0.08

References

Cai, T. T. and Ma, R. (2022). Theoretical foundations of t-sne for visualizing high-dimensional clustered data. *Journal of Machine Learning Research*, **23**(301), 1–54.

Krijthe, J. H. and van der Maaten, L. (2015). *Rtsne: T-Distributed Stochastic Neighbor Embedding using a Barnes-Hut Implementation*. R package version 0.15.

McInnes, L. *et al.* (2018). Umap: Uniform manifold approximation and projection for dimension reduction. *arXiv preprint arXiv:1802.03426*.

Melville, J. (2024). *uwot: The Uniform Manifold Approximation and Projection (UMAP) Method for Dimensionality Reduction*. R package version 0.2.2.9000.

Rousseeuw, P. J. (1987). Silhouettes: a graphical aid to the interpretation and validation of cluster analysis. *Journal of computational and applied mathematics*, **20**, 53–65.

Tibshirani, R. and Walther, G. (2005). Cluster validation by prediction strength. *Journal of Computational and Graphical Statistics*, **14**(3), 511–528.

Tibshirani, R. *et al.* (2001). Estimating the number of clusters in a data set via the gap statistic. *Journal of the Royal Statistical Society: Series B (Statistical Methodology)*, **63**(2), 411–423.

Van der Maaten, L. and Hinton, G. (2008). Visualizing data using t-sne. *Journal of machine learning research*, **9**(11).
