# Supplementary material for: Multidimensional scaling improves distance-based clustering for microbiome data
Source: Bioinformatics. 2025 Jan 28;41(2):btaf042. doi: 10.1093/bioinformatics/btaf042 (PMC11814494; doi:10.1093/bioinformatics/btaf042)
Supplement: btaf042_Supplementary_Data [file btaf042_supplementary_data.zip › btaf042_Supplementary_Data.zip!/28a82_supp_revision_MDS.pdf]

Response to Comments on "Multidimensional scaling improves distance-based clustering for microbiome data"

BIOINF-2024-1705

We thank the Associate Editor and the three Reviewers for their valuable comments, which have significantly improved our paper. We believe we have fully addressed the reviewers' concerns by thoroughly reconstructing the introduction and discussion sections, conducting additional simulations, clarifying both the simulation and real data results, and correcting typographical errors. Our detailed responses to the Reviewers' comments are provided below, with their comments shown in *italic text.*

Reviewer 1

Overall, the paper is well written.

Response: We appreciate your positive overall assessment of our paper

(R1-1) *Multidimensional scaling is one type of dimension reduction method, but there are many other methods available. If the key message is to perform dimension reduction before clustering, it would be valuable to explore whether other popular methods might achieve better performance. Could the authors compare their approach with PCA, kernel PCA, t-SNE, and UMAP to determine which dimension reduction method is most desirable?*

(R1-2) *A similar consideration applies to the clustering method. Have the authors explored other clustering approaches, such as mixture model approaches? Comparing these methods could provide insights into whether different clustering techniques might yield improved results.*

Response (R1-1) to (R1-2): We appreciate the reviewers' suggestion to broaden the scope of our paper. The original version focused on using MDS for dimensionality reduction followed by distance-based clustering. In the revised version, we explored combinations of MDS, PCA, t-SNE, and UMAP for dimensionality reduction, followed by distance-based clustering. The results are presented in the Supplementary Sections C.3 and D.2, with further discussion provided in the Discussion section. In particular, the following text describes how dimensionality reduction tools can be used as alternatives to MDS within our workflow: "Instead of using MDS as a dimensionality reduction tool, other techniques such as PCA, t-SNE (Van der Maaten and Hinton, 2008), and UMAP (McInnes *et al.*, 2018) can also be applied. In this comparison, steps 1–4 from our workflow were replaced with these techniques, while step 5—clustering in the reduced space—remained unchanged."

Overall, MDS outperformed PCA, t-SNE, and UMAP as a dimensionality reduction tool for microbiome data (see the figure below, corresponding to Figure S.2). The poor performance of PCA aligns with expectations, as it relies on Euclidean distance, which is unsuitable for compositional

data. A possible reason for the inferior performance of t-SNE and UMAP compared to MDS is that t-SNE and UMAP involve additional complex transformations beyond the original distance matrix during dimensionality reduction (see Supplementary Section C.3), while MDS directly operates on the Gram matrix of the distance. Similar discussion on these observations can be found in the Discussion Section.

Kernel PCA has been shown to be equivalent to Metric Multidimensional Scaling (mMDS), a generalized version of the classical MDS used in this study (Borg and Groenen, 2005; Williams, 2001). Given the demonstrated benefits of classical MDS in our approach, it is reasonable to hypothesize that other types of mMDS could further enhance clustering performance. While we know that at least one type of mMDS (Kernel PCA) is effective for denoising, whether other mMDS variants can achieve even better results remains an open question for future research. We have included a brief discussion of this point in the revised manuscript.

Additionally, while model-based clustering following dimensionality reduction represents an interesting avenue for exploration, our study specifically prioritized distance-based clustering. The rationale is that it is more natural to compare MDS with PAM to evaluate whether improvements arise specifically from the MDS step, as this is the only difference in the pipeline. After dimensionality reduction, the data is no longer compositional, making a Gaussian mixture model (GMM) the default choice for model-based clustering. However, directly applying GMM to microbiome data without dimensionality reduction would not be appropriate, as the compositional nature of the data violates key assumptions of Gaussian models. Therefore, GMM serves as a weak baseline for comparison, and the comparison with PAM offers a clearer assessment of the contribution of MDS to clustering performance. Therefore, exploring the integration of model-based clustering with dimensionality reduction methods like MDS was beyond the scope of this study. We have acknowledged this direction in the Discussion section as an area for future research.

Reviewer 2

(R2-1) *The authors demonstrated that the proposed method significantly improves the accuracy of distance-based clustering. However, in the simulation cases presented, the data dimensions are always smaller than the sample size, with 73 OTUs for the common lineage, 17 OTUs for the rare type, and 60 OTUs for the random type, while having 100 samples. It is important to discuss whether this method is suitable for high-dimensional datasets where the number of features (P) exceeds the number of samples (n). In high-dimensional scenarios, many methods experience performance degradation due to the curse of dimensionality. I am particularly interested in whether MDS can still enhance clustering performance under such conditions. Could the authors provide additional insights or simulation results to assess the effectiveness of the proposed approach in high-dimensional settings where $P > n$?*

Response: We are sorry for the confusion; the total number of OTUs is 800+, which is more than the sample size; 73, 17, and 60 OTUs are the OTUs with differential signals. Hence our simulation setting is already high-dimensional. We added a few sentences in the simulation section to clarify. In the discussion section, we also provided some discussion related to why MDS works.

(R2-2) *On line 44, page 10, the authors claim that distances incorporating phylogenetic tree information were more effective for the common and rare lineage cases, while distances without phylogenetic tree information were more effective for the random cases. However, in Figure 1 (DM – Nonrarefied-Random), the results show that UniFrac (with tree information) and G-UniFrac (with tree information) still achieve a higher ARI than BC (without tree information) and Jaccard (without tree*

DM - Nonrarefied - Common

PCA Result

	PS	GAP	SI	Oracle
PCA	0.02	0.41	0.532	0.629

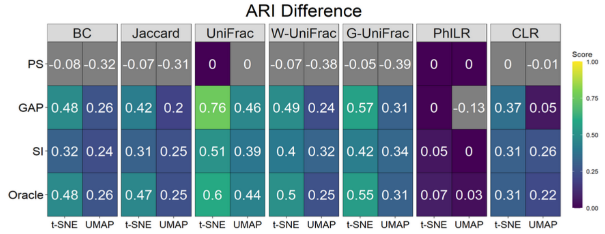

DM - Rarefied - Common

PCA Result

	PS	GAP	SI	Oracle
PCA	0.018	0.397	0.524	0.618

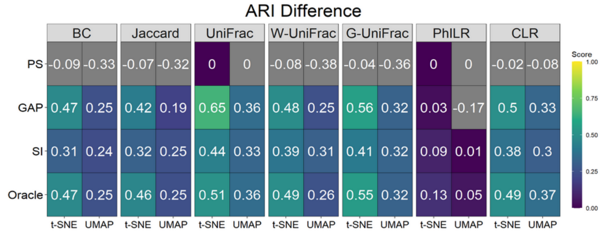

DM - Nonrarefied - Rare

PCA Result

	PS	GAP	SI	Oracle
PCA	0	0.008	0.228	0.368

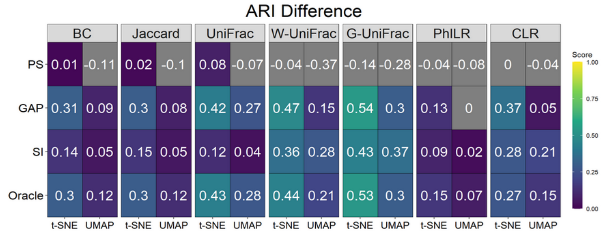

DM - Rarefied - Rare

PCA Result

	PS	GAP	SI	Oracle
PCA	0	0.007	0.229	0.36

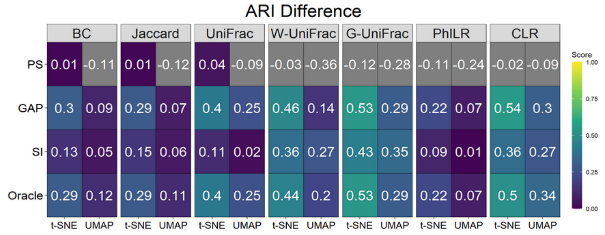

DM - Nonrarefied - Random

PCA Result

	PS	GAP	SI	Oracle
PCA	0.001	0.061	0.293	0.404

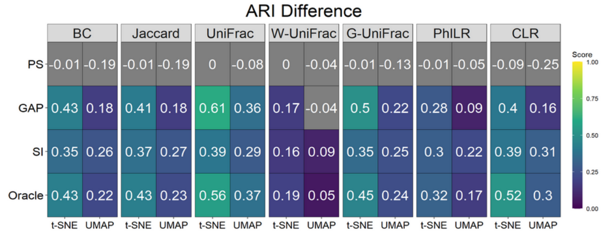

DM - Rarefied - Random

PCA Result

	PS	GAP	SI	Oracle
PCA	0	0.058	0.289	0.394

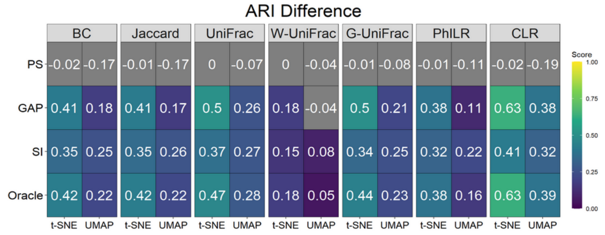

Figure S.2: Comparison of MDS, PCA, t-SNE, and UMAP in Scenario 1 using the Dirichlet Multinomial model as the generative framework. PCA results are presented using absolute ARI values, while t-SNE and UMAP results are shown as ARI differences relative to MDS (MDS - t-SNE or MDS - UMAP). Results for non-rarefied OTU data are displayed on the left, and rarefied OTU data on the right. In each heatmap, distance metrics are represented by columns, and cluster number selection methods by rows. Grey indicates scenarios where t-SNE or UMAP outperformed MDS.

information). This observation appears to contradict the stated trend, making it unclear. Could the authors clarify this inconsistency and provide further explanation to support their claim?

Response: We acknowledge the inconsistency in the observed trend across different scenarios, and we agree that our original statement was overly generalized. As such, we have removed the original claim from the text to avoid misinterpretation.

(R2-3) *In the simulation study, the Silhouette Index (SI) and Gap statistics performed well across both scenarios, while the predictive strength (PS) method was less effective than the other two. However, in the real data applications, the results were inconsistent: for the Martinez dataset, PS achieved perfect clustering, whereas for the Smits dataset, the ARI from the PS method was 0.00. These findings indicate that the choice of clustering evaluation method significantly affects the performance of the distance-based clustering approach. Could the authors provide recommendations or guidelines for users on how to choose among these methods based on different data characteristics or clustering scenarios?*

Response:

In our original simulation study, we primarily focused on scenarios with moderate to weak cluster separation. In response to the reviewer's suggestion, we conducted additional analyses under scenarios with well-separated clusters (see Section C.2 in the supplementary file). Specifically, we generated simulated data with a strong clustering signal by modifying Scenario 1 (Dirichlet multinomial model) based on the common lineage type described in the main text. As shown in the figure below (corresponding to Figure S.1 in the supplementary file), PS performed well in these cases, often achieving near-perfect clustering across most distance metrics. SI consistently performed well, while GAP showed poor performance for certain metrics. Additionally, we observed that GAP+MDS performed worse than GAP+PAM when paired with the Jaccard distance—a pattern consistent with the results observed in the Martinez dataset.

When geolocation serves as the true cluster label, the clustering signal is strong in the Martinez dataset but relatively weak in the Smits dataset. As a result, PS performs well for Martinez data but not for Smits data.

In the Discussion section, we recommend SI as the preferred cluster number selection method, as it consistently performs well across both weak and strong clustering signal scenarios, whereas GAP and PS exhibit inconsistent performance across different conditions.

The Results and Discussion sections have been updated to accurately reflect these findings.

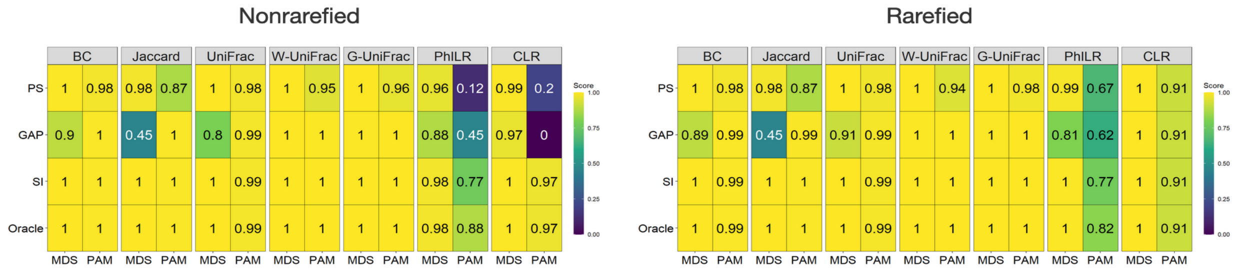

Figure S.1: ARI of different clustering methods for Scenario 1 of common taxa with DM as the generative model, the signal is stronger than those in the main text. The clustering results with non-rarefied OTU data are on the left, and those with rarefied OTU data are on the right. Within each heatmap, different distance metrics are displayed in columns with the cluster number selection methods displayed in rows. MDS and its PAM counterpart are placed side-by-side.

(R2-4) *Minor Comments: Line 42, page 10, under the random taxa Scenario 2, the ARI of DMM without rarefaction is reported as 0.44, and the ARI with rarefaction is reported as 0.45. However, in Figure 2, both values are shown as 0.45. Please clarify this discrepancy and ensure consistency between the text and the figure.*

Response:We apologize for the inconsistency. The correct ARI value for both cases (with and without rarefaction) is 0.45, as accurately shown in Figure 2. We have corrected the text to ensure consistency with the figure.

Reviewer 3

Overall, your work makes a significant contribution to improving microbiome data clustering.

Response: We appreciate your positive comments on our contribution.

(R3-1) *Minor: (1) "The OTU represents the species (known or unknown), and higher levels include class, genera, family, and kingdom." OTU may refer to species, class, genera, etc. (2) "P-dimensional vector for as the observed OTU for the i-th sample." Typo (3) Are any pre-processing steps needed before applying the method? (4) Page 5, line 34: clear up what total distance means - presumably sum of all distances of points to their medoid (5) Page 8, scenario 1: I'm getting a bit confused with the overuse of the word "set". In one instance it refers to the 20 lineages partitioned using PAM, in another instance it refers to a common lineage, rare lineage and 60 random OTUs. I think rewording these few sentences can make this scenario easier to understand.*

Response: (1) Thank you for the suggestion; we have revised the text to clarify the definition of OTU to ensure accuracy and consistency. (2) We apologize for the typo, and it has been corrected in the revised text. (3) There are no specific pre-processing steps required for our framework. We assume that the OTU counts have been converted to proportions, which is standard practice for all distance-based clustering methods. Therefore, we do not consider this step as a separate pre-processing requirement. While we investigated rarefaction as a pre-processing step, our method does not rely on rarefaction for effective performance. (4) We have clarified the meaning of "total distance" as the sum of all distances of points to their medoid as suggested. (5) We have rewritten the corresponding section in the simulation description to reduce the overuse of the term "set" and improve clarity and readability.

(R3-2) *While the methods and results are well described and convincing, the paper doesn't read well, because the introduction and discussion don't contextualize the project very well. The introduction starts at an awkward place (in the context of microbiome research) and doesn't motivate the need for this MDS project enough.*

(R3-3) *The discussion could talk more about why MDS works so well, and how authors might suggest users incorporate it into*

Response (R3-2) to (R3-3): Thank you for your valuable suggestion. We have rewritten the introduction to better motivate our study and enhanced the discussion section by elaborating on why MDS performs effectively in our framework. Additionally, we have conducted further simulations, as suggested by Reviewers 1 and 2, and incorporated the resulting insights into the discussion to provide greater clarity on the utility and application of MDS in clustering analysis.

References

Borg, I. and Groenen, P. J. F. (2005). *Modern Multidimensional Scaling: Theory and Applications*. Springer, New York.

McInnes, L. *et al.* (2018). Umap: Uniform manifold approximation and projection for dimension reduction. *arXiv preprint arXiv:1802.03426*.

Van der Maaten, L. and Hinton, G. (2008). Visualizing data using t-sne. *Journal of machine learning research*, **9**(11).

Williams, C. K. I. (2001). On a connection between kernel pca and metric multidimensional scaling. *Machine Learning*, **46**(1-3), 11–19.
